# C-755-02. Mismatch in Global Burn Care: Workforce and NGO Distribution versus Burden

**DOI:** 10.1093/jbcr/irag033.038

**Published:** 2026-04-06

**Authors:** Christopher J Fedor, Sarah M Tepe, Mare G Kaulakis, Francesco M Egro

**Affiliations:** University of Pittsburgh Medical Center Department of Plastic Surgery, Pittsburgh, PA; University of Pittsburgh Medical Center, Pittsburgh, PA; University of Pittsburgh Medical Center Mercy Burn Center, Pittsburgh, PA; University of Pittsburgh Medical Center, Pittsburgh, PA

## Abstract

**Introduction:**

Burn injuries remain a leading cause of preventable morbidity and mortality worldwide, particularly in low- and middle-income countries (LMICs). While the global burden of disease (GBD) framework reports burn-related deaths and disability-adjusted life years (DALYs), less is known about whether surgical workforce and non-governmental organization (NGO) presence are distributed in proportion to this need. We sought to quantify the mismatch between burn burden and burn care resources at the country level.

**Methods:**

We extracted 2021 GBD data on deaths (per 100 000) and DALYs (per 100 000) from fire, heat, and hot substances for all countries. Surgical workforce density (specialist surgeons per 100 000) was obtained from the World Bank/WHO database. Burn-related NGO presence was compiled from publicly available reports, with counts of NGOs per country. We created a standardized NeedScore (z-scores of DALY and death rates) and a CoverageScore (z-scores of workforce density and NGO count). The Mismatch Index was defined as NeedScore – CoverageScore; higher values represent greater need relative to coverage. We ranked countries by mismatch and generated comparative visualizations.

**Results:**

Among 183 countries with complete data, burn burden varied more than ten-fold. The highest age-standardized DALY rates were observed in Sub-Saharan Africa, while high-income countries reported <100 DALYs/100 k. Surgical workforce density was lowest in Africa (median 0.6 specialists per 100 k vs >40 per 100 k in high-income countries). NGO activity was concentrated in East Africa, but many high-burden countries reported few to none. The top five mismatch countries were Lesotho, Central African Republic, Somalia, Zimbabwe, and Eswatini, each with high DALY and death rates but <2 surgeons per 100 k and minimal or no NGO presence. Conversely, high-income countries such as Austria and Canada exhibited negative mismatch (low need, high coverage).

**Conclusions:**

Our global analysis demonstrates stark geographic disparities between burn burden and available care capacity. Countries in Sub-Saharan Africa carry the highest relative mismatch, reflecting high burn mortality and disability coupled with critical shortages in surgical workforce and NGO presence. Mapping these mismatches provides an evidence base for prioritizing educational partnerships, workforce development, and targeted NGO expansion in burn care. This open-data approach is scalable to other conditions relevant to plastic surgery and global health, highlighting opportunities for more equitable allocation of training and resources.

**Applicability of Research to Practice:**

As residents and physicians think about how they can expand their impact globally, it is important to have an assessment and understanding not only of where there is need for burn care, but also to contemplate which regions may still not receive the service they deserve.

**Funding for the study:**

N/A.

